# A randomized, single ascending dose safety, tolerability and pharmacokinetics study of NicaPlant® in aneurysmal subarachnoid hemorrhage patients undergoing clipping

**DOI:** 10.1016/j.bas.2023.102673

**Published:** 2023-09-18

**Authors:** Johannes Kerschbaumer, Christian Franz Freyschlag, Ondra Petr, Tiziana Adage, Joerg Breitenbach J, Lars Wessels, Stefan Wolf, Nils Hecht, Jens Gempt, Maria Wostrack, Matthias Gmeiner, Maria Gollwitzer, Harald Stefanits, Martin Bendszus M, Andreas Gruber, Bernhard Meyer, Peter Vajkoczy, Claudius Thomé

**Affiliations:** a Department of Neurosurgery, Medical University Innsbruck, Innsbruck, Austria; b BIT Pharma GmbH, Graz, Austria; c Department of Neurosurgery, Charité Berlin, Berlin, Germany; d Department of Neurosurgery, Klinikum Rechts der Isar, School of Medicine, Technical University Munich, Munich, Germany; e Department of Neurosurgery, Kepler University Hospital and Johannes Kepler University, Linz, Austria; f Department of Neuroradiology, University of Heidelberg, Heidelberg, Germany

**Keywords:** Aneurysm clipping, Aneurysmal subarachnoid hemorrhage, Cerebral vasospasm, Local delivery, Nicardipine

## Abstract

**Introduction:**

Aneurysmal subarachnoid hemorrhage (aSAH) is associated with high morbidity and mortality. Post-hemorrhagic vasospasm with neurological deterioration is a major concern in this context. NicaPlant®, a modified release formulation of the calcium channel blocker nicardipine, has shown vasodilator efficacy preclinically and a similar formulation known as NPRI has shown anti-vasospasm activity in aSAH patients under compassionate use.

**Research question:**

The study aimed to assess pharmacokinetics and pharmacodynamics of NicaPlant® pellets to prevent vasospasm after clip ligation in aSAH.

**Material and methods:**

In this multicenter, controlled, randomized, dose escalation trial we assessed the safety and tolerability of NicaPlant®. aSAH patients treated by clipping were randomized to receive up to 13 NicaPlant® implants, similarly to the dose of NPRIs previous used, or standard of care treatment.

**Results:**

Ten patients across four dose groups were treated with NicaPlant® (3–13 implants) while four patients received standard of care. 45 non-serious and 13 serious adverse events were reported, 4 non-serious adverse events and 5 serious adverse events assessed a probable or possible causal relationship to the investigational medical product. Across the NicaPlant® groups there was 1 case of moderate vasospasm, while in the standard of care group there were 2 cases of severe vasospasm.

**Discussion and conclusion:**

The placement of NicaPlant® during clip ligation of a ruptured cerebral aneurysm raised no safety concern. The dose of 10 NicaPlant® implants was selected for further clinical studies.

## Editor highlights

• Post-hemmorrhagic vasospasm with neurological deterioration is one major concern in this disease.
• Oral or intravenous nimodipine is standard of care after aSAH, but systemic side-effects often hinders adequate application.
• Local delivery of spasmolytics during angiography is short lasting and has not proven a benefit in terms of outcome.
• Local delivery over the critical period via prolonged released formulation may overcome the systemic side effects and have a beneficial effect in terms of outcome.

## 1 Introduction

Aneurysmal subarachnoid hemorrhage (aSAH) is associated with a high morbidity and mortality rate of up to 67% (Nieuwkamp, et al., 2009; Steiner et al., 2013) and a high burden on society (Kreiter, et al., 2002). The incidence reaches 5–9 per 100,000 population with a peak amongst those of 40–60 years. Aneurysm repair is readily achieved in most cases; recovery from the hemorrhage itself, however, is limited, so that only half of the survivors are able to live an independent life thereafter.

Inflammation after aSAH and cortical spreading depression (CSD) (Dreier, et al., 2009) may propagate a progressive tissue damage and post-hemorrhagic cerebral vasospasm is the major concern in this context, leading to ischemic events with associated delayed ischemic neurologic deficit (DIND) Currently, nimodipine is the only drug approved for consequences of posthemorrhagic vasospasm (Steiner, et al., 2013). This calcium channel blocker is administered orally or intravenously, despite limited evidence for intravenous administration (Geraldini, et al., 2022).

NicaPlant® (BIT Pharma GmbH) is a modified release formulation of the calcium channel blocker nicardipine, manufactured as rod shape and placed in close proximity to the major blood vessel walls at the time of microsurgical aneurysm treatment to ensure a prolonged release and local delivery of the active drug. The local tolerance of NicaPlant® was assessed preclinically using an open cranial window model. NicaPlant® induced dilatation of arterial vessels, which was not accompanied by increased vessel permeability or leukocyte–endothelial cell interaction due to the implant. Histology didn't reveal microglial activation or accumulation, nor structural neuronal changes (Bayerl, et al., 2019).

A similar drug composition named Nicardipine Prolonged-Release Implants (NPRI) was already administered under compassionate use in over 250 aSAH patients between 2002 and 2011 (Barth, et al., 2007; Kasuya, 2011; Kasuya et al., 2005; Kasuya et al., 2002; Krischek et al., 2007) when up to 12 implants were used, demonstrating good safety and tolerability profiles with no complications reported. In particular the dose of 10 implants showed significant reduction of the incidence of angiographic vasospasm and neurological deterioration (Barth, et al., 2007) emphasizing the need of further clinical investigations and the production of a nicardipine implant using an improved manufacturing process suitable for the market. In the current trial, we performed a phase IIA study to assess the safety and tolerability of the new formulation of NicaPlant®.

Based on the results of the clinical studies performed using the NPRI it is expected that the NicaPlant® efficacious dose range will be between 9 and 13 implants (36–52 mg modified release nicardipine). The safety and tolerability of up to 12 implants of the NPRIs has been previously demonstrated in aSAH patients. An initial dose of one quarter of this dose (3 implants, 12 mg nicardipine modified release) has been selected as a secure starting dose for NicaPlant ® safety and tolerability assessment. The trial registration number is EudraCT number 2016-004521-17.

## 2 Material and Methods

This was a randomized, controlled, single blinded and single ascending dose study in sequential aSAH patients (Hunt and Hess grade 1 to 4) undergoing aneurysm clipping.

Patients’ enrollment started on April 25th^,^ 2018, with the final patient completing the day 21 observation on January 10th^,^ 2019. NicaPlant® was administered by placement after microsurgical clipping of the ruptured aneurysm in proximity to all of the exposed cerebral blood vessels based on a predefined implantation scheme (Barth, et al., 2007). The study protocol included 4 cohorts with ascending doses of 3, 6, 10 or 13 implants containing 4 mg nicardipine each. Within the first two groups (3 and 6 implants), two patients received NicaPlant® and one standard of care control (SOC group); in the final two groups with 10 and 13 implants, three patients received NicaPlant® and one was assigned to SOC.

Between cohorts the study was placed on hold and an independent Data Safety Monitoring Board (DSMB) reviewed unblinded safety data collected up to day 21 post-treatment to ensure safety and tolerability and provide recommendation for study progression. The DSMB reviewed safety data and gave recommendation also in case of suspected unexpected serious adverse reaction (SUSARs).

Patients were randomized to the treatment groups within 48 h of aneurysm rupture. A member of the study team randomized patients in the interactive web response system receiving the randomization code for the patient. In the operation room, following aneurysm clip ligation, a member of the unblinded team verified the randomization code; if the patient was to receive NicaPlant® the implantation procedure went ahead.

During postoperative ICU care patients randomized to SOC received nimodipine orally, while patients randomized to NicaPlant® receive matching placebo tablets to avoid masking of possible systemic side effects of NicaPlant®, as well as to ensure blinding. No placebo implant was administered to the SOC group due to ethical reasons. Effort was put to maintain the blinding of all the personnel in the ICU and of those performing daily assessment.

Adverse events were meticulously recorded and followed up; daily assessed clinical parameters were shunt-dependent hydrocephalus, signs of bacterial meningitis and vital signs (blood pressure, pulse rate, respiratory rate and body temperature). Electrocardiogram, full blood count, urea and electrolytes, liver function test and C-reactive protein were monitored on alternating days (Table 1).

**Table 1:** Assessment of primary and secondary outcome parameters. AE: adverse events, DSA: digital subtraction angiography, CTA: Computed tomography angiography, ECG: echocardiogram, PK: pharmacokinetics, VS: vasospasm, GCS: Glasgow coma scale, TCD: transcranial doppler sonography, WFNS: World Federation of Neurosurgical Societies.

Visit →Time	Visit 1	Visit 2	Ongoing Assessments		Visit 3	Visit 4	Visit 5
	0–48 hrs after aSAH	Within 72 hrs of aSAH	to day 21 ± 1 post aneurysm		Day 8 ± 1	Day 14 ± 1	Day 21 ± 1
			Daily	Every 2nd Day			
Informed Consent	X						
DSA	X^i^				X^a^^,^^i^		
CTA	X^i^				X^i^		
CT Scan	X	X^b^				X	
Aneurysm Localization	X						
Inclusion/Exclusion	X						
Demographics/medical history	X						
Physical Examination	X						
Modified Rankin Scale	X						X
Pregnancy Test	X						
Adverse Event Reporting		X	X	X	X	X	X
Vital Signs^c^	X			X	X	X	X
ECG	X			X	X	X	X
Haematology & Serum biochemistry^d^	X			X	X	X	X
Randomization	X						
Clip Ligation & Implantation		X					
Post-Surgical Bacterial meningitis			X				
Shunt-dependent hydrocephalus			X				
PK Plasma^e^		X^f^			X	X	X
CSF Sampling^g^		X		X			
GCS	X		X				
WFNS	X						
Modified Fisher Scale	X						
Hunt & Hess Classification	X						
TCD^h^			X				
Recording of Nimodipine Administration			X				
Recording of Concomitant Medication	X	X	X	X	X	X	X

a A day 8 ± 1 angiogram will be performed even if the patient has no clinical or sonographic evidence of vasospasm. If the patient develops clinical or sonographic changes suggestive of vasospasm prior to day 8, an angiogram will be performed to confirm vasospasm, this will replace the one scheduled for day 8.

b Within 48 h post clip ligation.

c Blood pressure, pulse rate, respiratory rate and body temperature.

d FBC, U&Es, LFT, CRP.

e Visit 2, Visit 3, 4 and 5, blood collection - a single sample collected at the same time of the day as the time of implant.

f Visit 2 Plasma samples collected at time 0 (pre-implant), 2, 4, 6, 12, 24 h post implant.

g EVD/lumbar drain patients only; alternate days to day 21. At Visit 2 after 0 and 6 h post-clip ligation.

h TCD twice daily for evidence of vasospasm.

i Digital subtraction angiography (DSA) or CT angiography (CTA) at the discretion of the physician and according to the institutional protocol. DSA or CTA will only be performed if medically indicated.

Monitoring visits performed on-site to ensured GCP conformity and verified the source data and compliance with the protocol procedures.

Incidence of moderate or severe cerebral angiographic vasospasm (≥33% reduction in diameter in at least one vessel segment by comparison to preoperative angiography) was assessed by digital subtraction angiography (DSA) on day 8 ± 1. If doppler changes suggestive of vasospasm were noted (mean TCD velocity > 200 cm/s, daily increase of TCD velocity >50 cm/s; performed twice daily) prior to day 8 ± 1, DSA was conducted ahead of schedule.

Vasospasm -related morbidity and mortality was identified in case of DIND, cerebral infarctions or complications due to anti-vasospasm therapy or death. DIND was defined as a drop in Glasgow Coma Scale (GCS) or new neurologic deficits attributable to vasospasm.

Furthermore, the need for anti-vasospasm rescue therapy within the 21 days surveillance was recorded and a CT scan was performed on day 14 ± 1 for assessment of new cerebral infarcts compared to the CT scan performed on postoperative day 1. Angiograms and CT scans were analyzed by an independent neuroradiologist blinded to the treatment assignment (MB). The implants are not visible on the postoperative angiograms, even if in individual patients they can be suspected on the CT scan performed on postoperative day 1.

Blood sampling for pharmacokinetic assessment was carried out in every patient on the day of clip ligation at 0, 2, 4, 6, 12 and 24 h post clipping and on day 8, 14 and 21.

Cerebrospinal fluid (CSF) samples were collected at the time of clip ligation and 6 h after implantation and every second day in patients requiring an external ventricular drain (EVD) for medical reasons (Table 1).

Nicardipine levels were determined using a GLP validated method (pharm-analyt Labor GmbH, Baden, Austria). Frozen CSF and plasma samples were thawed, extracted and analyzed using an HPLC-MS/MS method. Nicardipine separation was performed with mobile phases composed of 0.1% formic acid in water (A) and 0.1% formic acid in acetonitrile (B). A linear gradient was used from 0 to 3.8 min, 10% → 50% B, followed by a 1min isocratic to 100% B gradient.

Nicardipine and D3-nicardipine HCl were used as reference and internal standard, respectively. For plasma the calibration range was 0.003–1,50 ng/ml, while for CSF was 0.5–250 ng/ml. Lower and higher concentrations represent the lower and upper limits of quantification.

Data were analyzed using SAS v9.4. Pharmacokinetic drug concentration over time profiles for individual subjects were used to derive the required PK curve parameters. These involved model-independent (non-compartmental) methods in specifically prepared SAS programs.

## 3 Results

### 3.1 Demographics

10 patients across 4 groups underwent active treatment with NicaPlant® implants (2 patients with 3 implants, 2 with 6 implants, 3 with 10 implants and 3 with 13 implants), whereas 4 patients were assigned to SOC. 10 female patients and 4 male patients were included. Source of the SAH was an aneurysm of the middle cerebral artery in 8 patients, 3 patients had an aneurysm of the anterior communicating artery or the anterior cerebral artery, 2 patients an aneurysm of the internal carotid artery and one patient a posterior communicating artery aneurysm. For further demographic data see Table 2.

**Table 2:** Demographics; *AcoA: anterior communicating artery, MCA: middle cerebral artery, ACA: anterior cerebral artery, ICA: internal carotid artery, PcoA: posterior communicating artery; HH: Hunt and Hess grade, VS: vasospasm; EVD: external ventricular drainage; i.a.: intra arterial nimodipine administration, p.o: oral nimodipine*.

Implants	Patient	Location	Fisher grade	HH	Infarcts^a^ (y/n)	VS	Rescue therapy	EVD (y/n)	Age (years)		Gender n(%)		Smoker status n(%)	
0	10,101	AcoA	2	2		severe	i.a.	n	mean	46.3	male	2(50)	yes	1(25)
0	10,103	MCA	2	2				n	min max	30 59	female	2(50)	no	3(75)
0	10,107	ACA	4	4		severe	i.a.	n						
0	20,101	ICA	2	2			i.a.^b^	n						
3	10,301	MCA	2	3				y	mean	59	male	2(100)	yes	2(100)
3	10,302	MCA	2	1		Unclear^c^	i.a.^c^	y	min max	59 59	female	0(0)	no	0(0)
6	10,102	MCA	4	4	y			y	mean	57	male	2(100)	yes	1(50)
6	10,303	PCoA	4	3				y	min max	52 62	female	0(0)	no	1(50)
10	10,104	AcoA	4	2				y	mean	65	male	2(66.7)	yes	1(33.3)
10	10,105	MCA	2	1				n	min max	61 67	female	1(33.3)	no	2(66.7)
10	10,106	MCA	2	1				n						
13	20,201	ICA	2	2				n	mean	39.7	male	2(66.7)	yes	1(33.3)
13	10,108	MCA	2	4	y	moderate	i.a	n	min max	29 47	female	1(33.3)	no	2(66.7)
13	20,202	MCA	4	3			p.o.^d^	n						

a Only new infarctions on the day 14 CT compared to the post-surgery CT are listed.

b Mild VS was diagnosed on site and intraarterial nimodipine was administered.

c Moderate VS was reported by the study site and rescue therapy performed.

d 60 mg nimodipine was orally given once as prophylaxis of vasospasm.

### 3.2 Safety

In total there were 45 non-serious adverse events reported during the study and 4 were assessed as having a probable or possible causal relationship to the investigational medical product, whereby only 2 of these patients received NicaPlant®. This included an episode of bradycardia (occurred in 1 subject who received NicaPlant®, 3 days after randomization) and fever (occurred in 1 patient, 6 days after randomization). There were 13 serious AEs reported (SAEs), of which 5 were assessed as having a probable to possible relationship to the implants (Table 3). No concerns about the safety of the tested implants were found by the DSMB or the investigators.

**Table 3:** SAEs by organ class.

System Organ Class		NicaPlant® (n = 10)	Control (n = 4)
Investigations	CSF culture positive	1	0
Nervous system disorders	Cerebral hematoma Cerebral vasoconstriction ICP increased Partial seizures	2 2 1 1	0 2 0 0
Respiratory, thoracic and mediastinal disorders	Respiratory distress	2	0
Vascular disorders	Cerebral artery occlusion	2	0

No deaths due to vasospasm or DIND were noted during the study observation period.

The centralized analysis identified two cases of severe vasospasm within the 4 patients in the SOC group and one case of moderate vasospasm in a NicaPlant® patient treated with 13 implants.

**Fig. 1:**
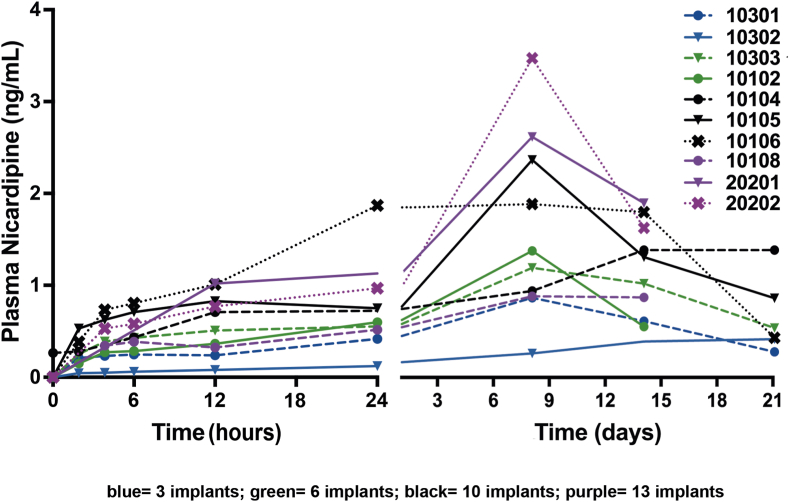
Plasma levels of nicardipine in the first 24 h after NicaPlant application in aSAH patients treated by aneurysm clipping (left) and during the 21 days study period (right) Individual patient data are presented.

Plasma levels of nicardipine were below the pharmacological active level (Cmax: 0.849 and 0.383 ng/ml for 3 implants, 1.38 and 1.19 ng/ml for 6 implants, 1.39, 2.41 and 1.91 ng/ml for 10 implants, 0.867, 2.67 and 3.56 ng/ml for13 implants; Fig. 1).

**Fig. 2:**
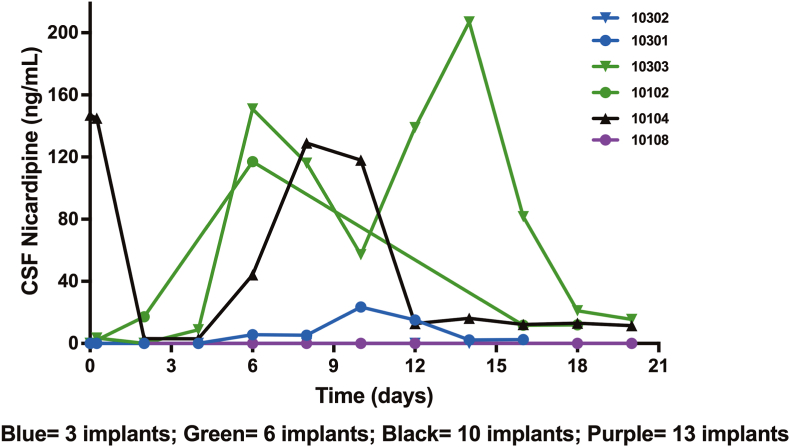
Cerebrospinal fluid (CSF) levels of nicardipine in the 6 aSAH patients receiving NicaPlant at aneurysm clipping and provided with external ventricular drain for medical reasons. Individual patient data are presented.

CSF samples were collected in 6 patients with an EVD resulting in drug concentration known to induce vasodilatation starting with 6 implants (Fig. 2). In one patient treated with 13 implants low CSF levels of nicardipine were measured, however this patient underwent revision surgery the day after implantation to remove a residual hematoma. This may have caused a partial loss of the investigational drug.

## 4 Discussion

In aSAH patients delayed cerebral ischemia due to vasospasm is a devastating condition occurring after the initial hemorrhage and tremendous efforts have been undertaken for its prevention or treatment given the high socioeconomical burden. The calcium channel antagonist nimodipine given orally is the only drug approved (Dorhout Mees, et al., 2007). Unfortunately, many patients are unable to assume it in the ICU setting and its application faces unfavorable side effect (mainly hypotension) especially in severe grade patients, causing treatment discontinuation (Sandow, et al., 2016) whilst not reaching therapeutic levels in CSF (Allen, et al., 1983; Petruk et al., 1988).

Intraarterial injection of nimodipine via catheterization result in an immediate ameliorating, but only temporary, effect and without improved clinical recovery (Biondi, et al., 2004; Grotenhuis et al., 1984; Liu et al., 2004; Numaguchi et al., 1997). To establish a stable vasodilatation, a continuous intraarterial nimodipine infusion (CIAN) was proposed. A recent review, however, linked this treatment to heparin-induced thrombocytopenia, atrial fibrillation or flutter, infections, acute kidney injury and systemic hypotension as most common side effects (Viderman, et al., 2021). Two studies suggested lower incidences of infarction and subsequent craniotomies with CIAN, but rates of tracheostomy and VP-shunting were increased ^4,32^. Therefore, its side effects as well as the longer and more intensive ICU treatment may counteract any beneficial effects (Kieninger, et al., 2018; Kieninger et al., 2019). As CIAN seems to address only macrovasospasm, it was concluded that may serve in well-selected patients with prolonged macrovasospasm (Hockel, et al., 2016).

An administration of microparticles containing nimodipine (EG-1962) via external ventricular drain has been investigated in a phase I/II dose-escalation study (Hänggi, et al., 2015) with fifty-four subjects randomized to ascending doses of EG-1962 compared to 18 patients treated with enteral nimodipine. The pooled data from all EG-1962 groups (100 mg–800 mg) suggested beneficial effect on the need for rescue therapy (24% vs 56%), delayed cerebral ischemia (31% vs 61%) and a higher rate of favorable clinical outcome. Disappointingly, the subsequent the phase III trial was stopped at the interim futility analysis.

Local application of the calcium channel blockers nicardipine, a calcium channel blocker approved in Japan for aSAH, via implants known as “Nicardipine Prolonged Release Implants” has been investigated after microsurgical clipping in more than 250 patients in Japan and Germany in the context of compassionate use (Barth, et al., 2007; Kasuya et al., 2005; Kasuya et al., 2002; Schneider et al., 2011). A favorable safety and tolerability profile has been described without signs of neuronal toxicity or chemical meningitis. A significant reduction of cerebral vasospasm and a reduced rate of DCI concomitant with a more favorable clinical outcome and reduced mortality was also reported in patients treated with 10 implants (Barth, et al., 2007).

BIT Pharma has developed NicaPlant® a comparable formulation that can be placed during surgical clipping of a ruptured aneurysm allowing delivery of nicardipine locally, to eliminate or reduce systemic side effects and maximize efficacy.

Preclinical data in mice have shown that NicaPlant® pellets release nicardipine over a period of 21 days with 80% release by day 15 and an almost linear dissolution. Vasoactive vessel diameter dilatation and no reduction of neuronal density or inflammatory reaction due to the implants was noted in the preclinical mouse model setting (Bayerl, et al., 2019).

In the present trial we aimed to assess safety and tolerability of the implants in humans.

According to our data 6 to 10 implants seem suitable in terms of CSF levels reached, while 13 implants did not add further benefits, but may require further dissection of the basal cisterns to create sufficient room for placement.

Most importantly, the administration of up to 13 implants did not raise the nicardipine plasma levels, thus avoiding systemic side effects.

There were 13 serious adverse events reported in the present study, of which 5 were assessed as having a probable to possible relationship to NicaPlant in 2 patients. One patient with 10 implants developed an intracerebral hematoma on day 14 after clipping of an MCA aneurysm, with a recurrent hematoma on day 28. The hematoma was associated with focal motor seizures and necessitated antiepileptic treatment. Even if an irritation of the brain by the implants cannot be excluded, the semiology of the epileptic seizures cannot be directly linked to the location of the implants. Both the hematoma and its recurrence were evacuated after DSA, which failed to reveal aneurysm recurrence or vessel alteration. Thus, the cause of the hemorrhages remains unclear.

One patient developed an occlusion of an M2 branch during aneurysm repair, which was noted in the intraoperative ICG angiography. Patency through the branch was successfully restored via thrombectomy immediately after clipping of the aneurysm, but a re-occlusion occurred on postoperative day 4. The patient developed an increased ICP on the day after clipping, which was interpreted as being probably related to the treatment. These 2 SUSARs were rated as probable related, even though also the large initial hematoma in the Sylvian fissure and the small infarctions around the hematoma in the postoperative CT could at least partially explain the elevated ICP noted. The re-occlusion of the M2 branch in the control DSA cannot be directly linked to the treatment, as the initial thrombosis is naturally associated with a high chance of recurrence even after successful recanalization.

Of the 45 non-serious adverse events documented during the trial, only 4 were assessed as having a probable or possible causal relationship to the treatment, whereas only 2 of these patients received NicaPlant.

Although not powered for efficacy, the incidence of moderate or severe vasospasm with its related morbidity and mortality, as well as cerebral infarcts on CT were assessed to obtain preliminary data. Treatment with NicaPlant suggests an overall reduced incidence of vasospasm compared to the SOC group, this will need to be confirmed in a subsequent clinical trial.

These data, together with the preclinical data, supported an orphan medicinal product designation for nicardipine (NicaPlant®) in the treatment of non-traumatic subarachnoid hemorrhage by the European Medicinal Agency.

## 5 Conclusion

In conclusion, the placement of NicaPlant® implants after microsurgical clipping raised no safety concerns. According to the pharmacokinetic data, preliminary efficacy results and usability, the dose of 10 implants has been selected for further clinical studies to investigate clinical efficacy (EudraCT number 2017-005159-10).

## Funding

The trial was partly supported by the Austrian research promotion agency project 860144.

## Declaration of competing interest

TA is Chief Scientific Officer at BIT Pharma; BJ is Chief Executive Officer, co-founder and shareholder at BIT Pharma; VP is consultant and shareholder at BIT Pharma.

The other authors declare no conflict of interests.

## References

[bib1] Allen G.S. (1983). Cerebral arterial spasm--a controlled trial of nimodipine in patients with subarachnoid hemorrhage. N. Engl. J. Med..

[bib3] Barth M. (2007). Effect of nicardipine prolonged-release implants on cerebral vasospasm and clinical outcome after severe aneurysmal subarachnoid hemorrhage: a prospective, randomized, double-blind phase IIa study. Stroke.

[bib4] Bayerl S.H. (2019). In vitro and in vivo testing of a novel local nicardipine delivery system to the brain: a preclinical study. J. Neurosurg..

[bib5] Biondi A. (2004). Intra-arterial nimodipine for the treatment of symptomatic cerebral vasospasm after aneurysmal subarachnoid hemorrhage: preliminary results. AJNR Am. J. Neuroradiol..

[bib9] Dorhout Mees S.M. (2007). Calcium antagonists for aneurysmal subarachnoid haemorrhage. Cochrane Database Syst. Rev..

[bib10] Dreier J.P. (2009). Cortical spreading ischaemia is a novel process involved in ischaemic damage in patients with aneurysmal subarachnoid haemorrhage. Brain.

[bib11] Geraldini F. (2022). A comparison between enteral and intravenous nimodipine in subarachnoid hemorrhage: a systematic review and network meta-analysis. Neurocritical Care.

[bib12] Grotenhuis J.A. (1984). Intracarotid slow bolus injection of nimodipine during angiography for treatment of cerebral vasospasm after SAH. A preliminary report. J. Neurosurg..

[bib14] Hänggi D. (2015). Newton: nimodipine microparticles to enhance recovery while reducing toxicity after subarachnoid hemorrhage. Neurocritical Care.

[bib16] Hockel K. (2016). Long-term, continuous intra-arterial nimodipine treatment of severe vasospasm after aneurysmal subarachnoid hemorrhage. World Neurosurg.

[bib18] Kasuya H. (2011). Clinical trial of nicardipine prolonged-release implants for preventing cerebral vasospasm: multicenter cooperative study in Tokyo. Acta Neurochir. Suppl..

[bib19] Kasuya H. (2005). Application of nicardipine prolonged-release implants: analysis of 97 consecutive patients with acute subarachnoid hemorrhage. Neurosurgery.

[bib20] Kasuya H. (2002). Efficacy and safety of nicardipine prolonged-release implants for preventing vasospasm in humans. Stroke.

[bib21] Kieninger M. (2018). Side effects of long-term continuous intra-arterial nimodipine infusion in patients with severe refractory cerebral vasospasm after subarachnoid hemorrhage. Neurocritical Care.

[bib22] Kieninger M. (2019). Incidence of arterial hypotension in patients receiving peroral or continuous intra-arterial nimodipine after aneurysmal or perimesencephalic subarachnoid hemorrhage. Neurocritical Care.

[bib23] Kreiter K.T. (2002). Predictors of cognitive dysfunction after subarachnoid hemorrhage. Stroke.

[bib24] Krischek B. (2007). Nicardipine prolonged-release implants for preventing cerebral vasospasm after subarachnoid hemorrhage: effect and outcome in the first 100 patients. Neurol. Med.-Chir..

[bib25] Liu J.K. (2004). Efficacy of multiple intraarterial papaverine infusions for improvement in cerebral circulation time in patients with recurrent cerebral vasospasm. J. Neurosurg..

[bib27] Nieuwkamp D.J. (2009). Changes in case fatality of aneurysmal subarachnoid haemorrhage over time, according to age, sex, and region: a meta-analysis. Lancet Neurol..

[bib28] Numaguchi Y. (1997). Repeat intra-arterial papaverine for recurrent cerebral vasospasm after subarachnoid haemorrhage. Neuroradiology.

[bib29] Petruk K.C. (1988). Nimodipine treatment in poor-grade aneurysm patients. Results of a multicenter double-blind placebo-controlled trial. J. Neurosurg..

[bib30] Sandow N. (2016). Nimodipine dose reductions in the treatment of patients with aneurysmal subarachnoid hemorrhage. Neurocritical Care.

[bib31] Schneider U.C. (2011). The use of nicardipine prolonged release implants (NPRI) in microsurgical clipping after aneurysmal subarachnoid haemorrhage: comparison with endovascular treatment. Acta Neurochir..

[bib32] Steiner T. (2013). European Stroke Organization guidelines for the management of intracranial aneurysms and subarachnoid haemorrhage. Cerebrovasc. Dis..

[bib33] Viderman D., Sarria-Santamera A., Bilotta F. (2021). Side effects of continuous intra-arterial infusion of nimodipine for management of resistant cerebral vasospasm in subarachnoid hemorrhage patients: a systematic review. Neurochirurgie.

